# The association of maternal food quality score (FQS) with breast milk nutrient content and antioxidant content of infant urine: a cross-sectional study

**DOI:** 10.1186/s12884-023-05400-3

**Published:** 2023-02-24

**Authors:** Samira Karbasi, Malihe Mohamadian, Mohsen Naseri, Mohammad Yahya Hanafi-Bojd, Zahra Khorasanchi, Negar Morovatdar, Asghar Zarban, Afsane Bahrami, Gordon A. Ferns

**Affiliations:** 1 Department of Molecular Medicine, School of Medicine, Cardiovascular Diseases Research Center, Birjand University of Medical Sciences, Birjand, Iran; 2 Cellular and Molecular Research Center, Department of Molecular Medicine, Birjand University of Medical Sciences, Birjand, Iran; 3 Department of Pharmaceutics and Pharmaceutical Nanotechnology. School of Pharmacy, Birjand University of Medical Sciences, Birjand, Iran; 4 Department of Nutrition, School of Medicine, Mashhad University of Medical Sciences, Mashhad, Iran; 5 Clinical Research Development Unit, Faculty of Medicine, Imam Reza Hospital, Mashhad University of Medical Sciences, Mashhad, Iran; 6 Cardiovascular Diseases Research Center, Birjand University of Medical Sciences, Birjand, Iran; 7 Clinical Biochemistry Department, Faculty of Medicine, Birjand University of Medical Sciences, Birjand, Iran; 8 Clinical Research Development Unit of Akbar Hospital, Mashhad University of Medical Sciences, Mashhad, Iran; 9 Brighton and Sussex Medical School, Division of Medical Education, Falmer, Brighton, BN1 9PH Sussex UK

**Keywords:** Breast milk, Antioxidant activity, Food quality, Lactating mothers, Infant urine

## Abstract

**Background:**

Breast milk (BM) is a complex fluid with a variable composition within women over time and between women in the population. The BM compositional differences are likely to be partly due to maternal dietary patterns. This study aimed to evaluate food quality score (FQS) in lactating mothers and its association with quality indicators of BM and antioxidant content of infant urine.

**Methods:**

This cross-sectional study was undertaken in 350 lactating women aged 20 to 35 years. Data on dietary intake was collected using a validated food frequency questionnaire (FFQ) containing 65 food items. The FQS was calculated by integrating the scores obtained from healthy and unhealthy food groups. Subjects were categorized according to FQS adherence, with the greatest adherence being allocated to the third tertile and those with the lowest FQS in the first tertile. Antioxidant activity of the BM and infant urine samples was assessed using the Ferric reducing antioxidant power (FRAP), 2, 2′-diphenyl-1-picrylhydrazyl (DPPH), thiobarbituric acid reactive substances (TBARs), and Ellman’s assay. The total content of BM protein, calcium, and triglyceride was measured using standard biochemical kits.

**Results:**

BM from mothers from the third tertile of FQS contained significantly higher DPPH, thiol, calcium, and protein levels compared to BM from those in the lowest tertile (*p*˂0.05). Infant urinary DPPH and FRAP was also significantly higher in the highest tertile vs. the lowest tertile (*p*˂0.05).

**Conclusion:**

High maternal adherence to the FQS was associated with a high BM quality and antioxidant content of infant urine.

## Introduction

Breast milk (BM) feeding is recommended by the World Health Organization (WHO) as the exclusive source of infant nutrition during the first six months of life followed by continued breastfeeding for at least two years [1]. Both in terms of nutritional composition and non-nutritious bioactive factors, BM secures optimal health and development of infants [2]. Breastfeeding is a decisive factor in reducing the risk of Sudden Infant Death Syndrome (SIDS) [3] as well as long-term mortality and morbidity from infectious diseases [4], cardiovascular disease (CVD) [5], gastrointestinal disorders [6, 7], metabolic diseases [8], allergic pathologies [9], and cognitive impairment [10] in infants. BM contains macronutrients (proteins, carbohydrates, and lipids), micronutrients (vitamins and minerals), non-nutrient bioactive compounds (antioxidants, growth factors, hormones, and prebiotics), and components protecting against infection (immunoglobulin A [IgA], lactoferrin, oligosaccharides, and lysozyme) [11]. Infants often struggle with the challenges of oxidative stress caused by the rapid transition from intrauterine to extrauterine milieu with much more oxygen [12]. Oxidative stress, defined as the homeostatic imbalance between oxygen-derived metabolites (predominantly reactive oxygen species [ROS]) and antioxidant defense systems, is responsible for a wide spectrum of infant diseases [13]. It is well known that antioxidant components of BM, including superoxide dismutase, glutathione peroxidase, catalase, vitamins (A, C, and E), carotenoids, α-tocopherol, and thiols protect infants against ROS-related conditions [12, 14]. The higher content of urinary antioxidant biomarkers in breastfed infants compared to formula-fed infants is partial evidence of these protective effects of the BM [15]. However, BM is a complex and dynamic fluid with a variable composition between mothers and within populations. The BM compositional differences are due to several maternal, infant, and environmental factors such as lactation stage, term/preterm delivery, and dietary patterns [16].

Evidence suggests that total antioxidant capacity (TAC), and the content of fatty acids (FAs) and vitamins A, C, B-6, and B-12 in BM are closely related to maternal nutrition during pregnancy [17, 18]. Since the assessment of single nutrient intakes does not accurately reflect the overall quality of the diet, dietary scores that take the whole diet into account, like the Food Quality Score (FQS) have been developed. Nutrient intake assessments do not require a database or software, and food-based scores are easily adjusted for clinical use [19]. FQS is usually determined by integration of the different food groups that fall into two categories: healthy and unhealthy [20]. FQS has previously been assessed in a limited number of studies where its association with the risk of metabolic syndrome [21], coronary heart disease [22], CVD [20], and breast cancer [23] was evaluated. Although many studies have been conducted on the impact of maternal dietary patterns such as vegetarianism, and a Mediterranean diet on BM composition among different populations [24–26], no study has focused on the relationship between FQS and antioxidant content of BM and infant urine. Therefore, this study aimed to investigate the FQS in lactating mothers and its association with quality indicators of BM and infant urine including TAC and content of some micro and macronutrients.

## Material and methods

### Study design and participants

**Fig. 1:**
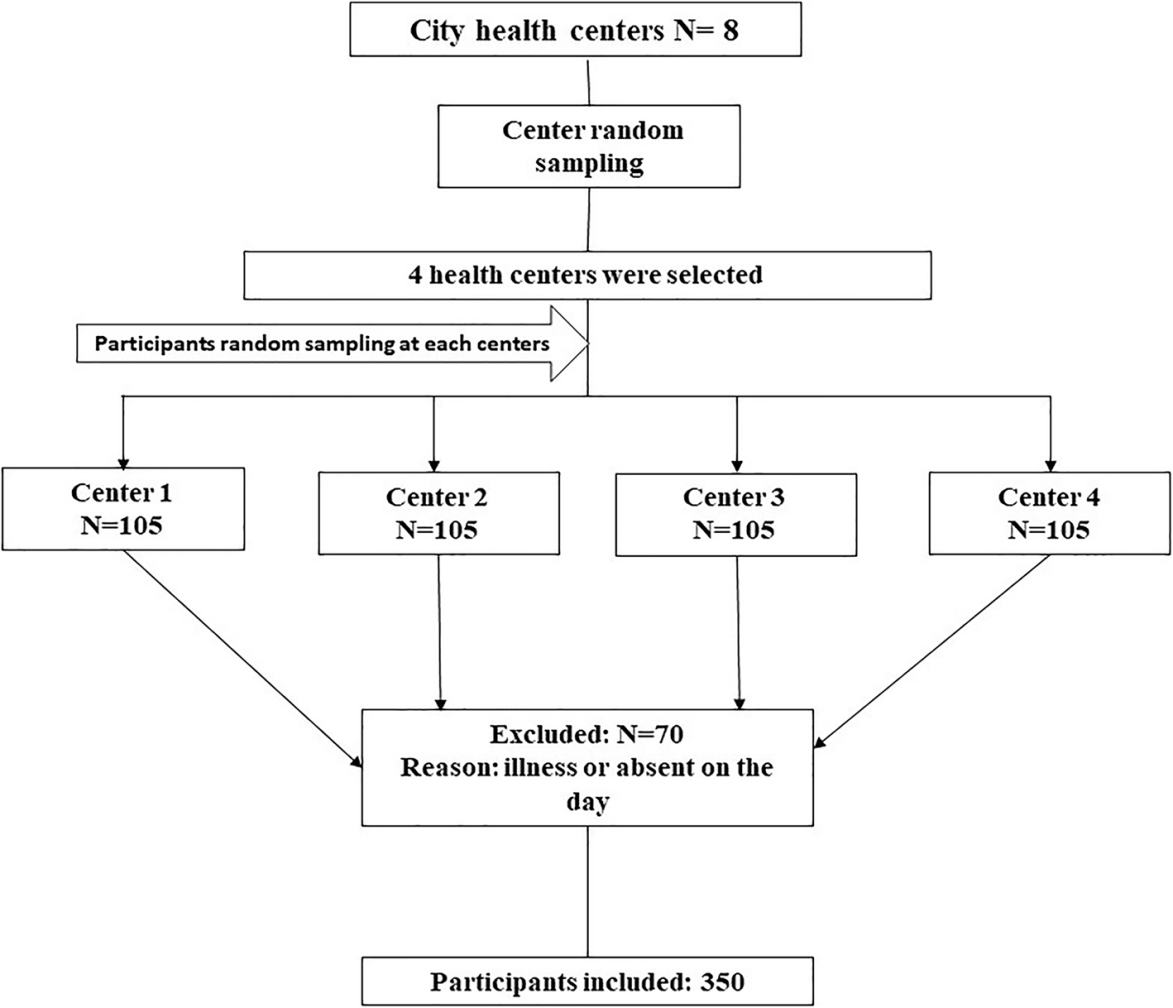
Enrollment flow chart of lactating women

In this cross-sectional study, 350 breastfeeding mothers aged between 20 to 35 years were recruited from four healthcare centers in southern Khorasan, Birjand, Iran in 2021. Participant’s inclusion was performed by a cluster random sampling method. Written informed consent was taken from subjects prior to recruitment. All participants had infants between the ages of 1 and 6 months, with no history of chronic diseases or medication intake in the last six months. The sample size for the study was calculated with 80% power using the PASS ver.11 using a mean±sd of Urinary MDA (μmol TBARs/mg Cr) in the first and last tertiles of DASH diet and α=0.05 [27]. According to this calculation and regards to design effect (Deff) =1.5 for cluster random sampling design, 350 participants were calculated for all 4 clusters; nevertheless, 420 participants (105 participants for each cluster) were initially recruited to account for data availability and for possible exclusions and drop outs. Fig. 1 shows a flow chart of the recruitment and follow-up numbers of lactating women in the study.The Ethics Committee of Birjand University of Medical Sciences approved the study. Each mother was requested to provide two samples of BM in 20 ml volumes expressed from the primary breastfeeding at the beginning of the day. In addition, a 10 ml urine sample was taken from each infant at by urine bag. Samples were collected in sterile tubes and stored quickly at -80 °C until processing.

### Dietary assessments

To record the dietary data of the participants, a 65-semi-quantitative food frequency questionnaire (FFQ) was completed. A trained dietician asked mothers to rate their frequency of food consumption for each item over the course of the previous year as daily, weekly, monthly, sometimes or never. The reliability and validity of the FFQ has previously been reported in the Iranian population [28]. After collecting participants’ information about the type, size, and frequency of consumption of each food item, the average daily consumption (gr/day) was calculated for reported foods using household scales. We used Nutritionist IV software (version 7.0; N-Squared Computing, Salem, OR) rectified for Iranian food ingredients for nutrient and energy intake assessment.

#### FQS determination

Food quality scoring was performed by the scale developed by Fung et al*.* [22]. FQS ingredients include vegetables, fruits, whole grains, nuts and legumes, yogurt, coffee as healthy foods as well as refined grains, sugar-sweetened beverages, desserts and ice cream, red and processed meats, potato and potato chips, and fried food consumed outside the home as unhealthy foods. We then classified the participants' intake into deciles. A value between 1 and 10 was assigned to each healthy component. For unhealthy components, a reverse scoring process (values between 10 and 1) was assigned. Finally, the total FQS (in the range of 14 to 140) was calculated by summing all the scores obtained for each participant, so that a higher score indicates a healthier diet.

### Measurement of antioxidant activity

Total antioxidant status of BM and infant urine samples was determined using four different assays: the Ferric reducing antioxidant power (FRAP), 2, 2′-diphenyl-1-picrylhydrazyl (DPPH), Thiobarbituric acid reactive substances (TBARS), and Ellman’s assay as described below.

#### FRAP assay

The assay was based on the method of Benzie and Strain [29], in which the reduction of the ferric-tripyridyl-s-triazine (TPTZ) complex in working FRAP reagent to the ferrous-TPTZ followed by the formation of an intense blue color that can be measured by the adsorption amount at 593 nm [30]. Ten µL of each BM or infant urine sample (1:10 diluted), standard (FeSO4) or blank (for each milk sample, a blank sample was used to remove milk turbidity) was mixed with 250 µL of the working FRAP reagent. Following incubation of the reaction mixture at 37 °C for 10 min, its absorption was measured using a spectrophotometer at 593 nm. All tests were run in duplicate and the results are reported in µmol/L.

#### DPPH assay

A modification of the method proposed by Brand-Williams et al. was applied [31]. This is based on the reduction of violet DPPH radical to a stable pale-yellow molecule (1, 1- diphenyl-2- picrylhydrazine) and subsequent spectrophotometric measurement of the residual DPPH radical [32]. For BM, after adding 50 μl of the samples to 950 μl of DPPH solution and incubating at room temperature for 10 min, the resulting mixture was centrifuged at 3000 g for 3 min and the supernatants were assessed spectrophotometrically at 517 nm. Each infant urine sample was centrifuged at 3000 g for 3 min and diluted 1/10, followed by adding 20 μl of each sample to 250 μl of DPPH solution. Using an methanolic DPPH solution (100 mM) as a control, and adsorption assessment at 517 nm. Percent of DPPH radical scavenging activity was calculated as: [(absorbance of the control – absorbance of the sample)/absorbance of the control] *100.

Each test was repeated twice, and the results were reported in µmol Trolox equivalent /L.

#### TBARs assay

Using the method developed of Placer et al. [33], the reaction of thiobarbituric acid (TBA) with malondialdehyde (MDA) leaves a pink complex (TBARs) that can be detected spectrophotometrically. For the assay, 100 μl of each sample (BM and urine) was mixed with 1 ml of TBA/trichloroacetic acid/HCl reagent, and the mixture was heated in a water bath for 20 min. After precipitating the TBARs adducts with 1 ml of N-butanol and subsequent dissolution in water, the fluorescence was read at excitation and emission wavelengths of 515 and 553 nm, respectively. The results (μmol TBARs/L) were obtained using a standard curve using different concentration of 1.1.2.2 tetramethoxy propane as standard.

#### Ellman’s assay

To monitor the free thiol groups in BM samples, 5,5’-dithio-bis-(2-nitrobenzoic acid) (DTNB) or Ellman’s reagent was used [34]. Briefly, 50 µl of BM samples was added to 1 ml of Tris/EDTA buffer and 50 µl of 10 mM DTNB solution and after incubation period, 650 µL N-butanol was added. The mixture was centrifuged for 5 min at 3000 g. The absorbance was measured at 412 nm, and the net adsorption was calculated by subtracting the apparent absorbance from the absorbance of a DTNB blank (which contained methanol instead of the samples). The standard curve was prepared using reduced glutathione and the results were expressed as μmol/L [35].

### Biochemical assessments

The nutrient content of BM samples was also assessed using standard biochemical kits (Pars Azmoon, Tehran, Iran) for the total levels of protein, calcium, and triglyceride. All photometric analyses were performed at 37 °C using a plate reader (EpochTM, BioTek, Winooski, VT, USA). To evaluate all absorbance information, monochromatic readings were taken [36].

### Anthropometric and demographic assessment

A trained nurse evaluated information including mother age, mother systolic blood pressure (SBP), mother dyastolic blood pressure (DBP), mother body mass index (BMI) infant age, and infant head circumference (cm). Each parcipitant's height and weight were assessed using standard procedures and the BMI [weight (kg)/height (m^2^)] was estimated. With a tape measure, the height and circumference of the infant's head were measured to the nearest millimeter. To document the weight to the nearest 0.1 kg, electronic scales were used. SBP and DBP assessments after sitting and resting were performed repeatedly using a mercury sphygmomanometer for 25 minutes period, and the average of the assessments was documented.

### Statistical analysis

The calculated FQS were classified into tertiles (T1: low adherence, T3: high adherence). To assess the variables’ normality, the Kolmogorov-Smirnov analysis was employed. To study continuous variables (BM and infant urinary) with normal distribution among tertiles, a one-way ANOVA test was used. Linear regression methods were used to calculate adjusted β estimates and 95% confidence interval (CIs) were also undertaken to specify the association between the maternal FQS with nutrient content of BM and infant urinary. Regression models were adjusted for BM samples: for maternal age, BMI, energy intake, and for infant urine:for infant age. All statistical analyses were performed using the statistical package for social sciences (SPSS, version 16.0) software, with a significance level of *P* <0.05.

## Results

### Demographic and anthropometric of the participants in the different tertiles of adherence to the FQS (Table 1)

**Table 1:** Demographic, anthropometric and clinical characteristics of the participants in different tertiles of the adherence to the food quality score (FQS)

Variables	**T1** 118(33.71%)	**T2** 122(34.85%)	**T3** 110(31.42%)	*P* value^a^
Mother Age (y)	29.6 ± 5.4	29.2 ± 6.5	29.5 ± 6.1	0.85
Mother SBP (mmHg)	102 ± 1.0	101 ± 0.8	100 ± 0.9	0.58
Mother DBP (mmHg)	82 ± 0.6	74 ± 0.8	86 ± 0.2	0.68
Mother BMI (Kg/m^2^)	26.4 ± 5.1	26.9 ± 1.7	23.3 ± 3.0	0.39
Infant Age (d)	128 ± 37.7	129.1 ± 35.7	139.7 ± 40.4	0.64
Infant head circumference (cm)	39.2 ± 4.7	39.1 ± 3.6	40.7 ± 3.6	0.22

Data presented as Mean ± SD

T1 represents low adherence and T3 a high adherence with a FQS

Body mass index (BMI), Systolic blood pressure (SBP), Diastolic blood pressure (DBP)

^a^*p*-value from analysis of the variance (ANOVA)

The 350 women aged 29.5±5.9 years were divided into 3 groups based on the tertiles of their FQS: T1 was in the lowest tertiles (lowest adherence; *n*=118), T2 was in the second (*n*=122), and T3 was in the highest (higher adherence; *n*=110). There was no significant relationship between the participants' general anthropometric and demographic data including mother age, mother SBP, mother DBP, mother BMI, infant age and infant head circumference for participants in the lowest (T1) and greatest (T3) tertiles of FQS adherence (*P*>0.05). All the results were normal according to Kormogorov Smirnov (*P*>0.05).

### Comparison of dietary intakes of participants between highest and lowest tertiles of the FQS (Table 2)

**Table 2:** Comparison of dietary intakes of participants between highest and lowest tertiles of the adherence to the FQS

Variables	Tertiles of FQS			*P*-value
	**T1** 118(33.71%)	**T2** 122(34.85%)	**T3** 110(31.42%)	
Vitamin B6 (mg/day)	1.61 ± 0.77	1.11 ± 0.40	1.17 ± 0.41	0.18
Vitamin B12 (μg/day)	2.51 ± 1.41	2.65 ± 1.31	3.01 ± 1.16	** < 0.001**
Vitamin C (mg/day)	151.6 ± 121	159.6 ± 101	264.1 ± 243	**0.005**
Vitamin D (μg /day)	1.37 ± 8.3	1.15 ± 6.2	1.32 ± 3.6	0.78
Vitamin E (mg/day)	26.9 ± 44.4	38.3 ± 27.2	44.6 ± 38.6	**0.04**
Thiamine (mg/day)	1.69 ± 0.76	1.13 ± 0.28	1.18 ± .65	0.11
Vegetables (g/day)	100.1 ± 137.1	104.1 ± 125.1	114.6 ± 116.8	** < 0.001**
Fruits (g/day)	106.7 ± 28.3	120.1 ± 53.4	168.8 ± 23.6	** < 0.001**
Legumes and nuts (g/day)	14.5 ± 53	19.8 ± 27	23.5 ± 63	** < 0.001**
Whole grains (g/day)	105.9 ± 25.5	107.5 ± 27.4	117.6 ± 42.3	**0.01**
Yogurt (g/day)	192.2 ± 55.7	220.2 ± 49.1	225.4 ± 47.1	** < 0.001**
Sugar sweetened beverage (g/day)	25.2 ± 86.4	20.1 ± 87.0	16.2 ± 70.3	** < 0.001**
Red meat (g/day)	19.5 ± 47	18.2 ± 38	16.7 ± 32	** < 0.001**
Processed met (g/day)	2.39 ± 4.7	2.17 ± 5.3	2.11 ± 3.2	** < 0.001**
Refined grains (g/day)	168.3 ± 19.1	113.5 ± 34.4	105.4 ± 23.7	** < 0.001**
Desserts and ice cream (g/day)	195.2 ± 32.1	190.1 ± 19.1	188.5 ± 19.6	**0.006**
Potato (g/day)	181.6 ± 53.4	158.1 ± 53.9	144.1 ± 51.9	** < 0.001**
Potato chips (g/day)	198.3 ± 22.2	196.0 ± 19.8	194.2 ± 13.2	0.42
Coffee (g/day)	191.3 ± 37.4	198.2 ± 13.4	200.0 ± 53.5	0.54
Fried food from outside the home (g/day)	12.9 ± 7.5	12.1 ± 5.1	11.5 ± 6.8	** < 0.001**

Data presented as Mean ± SD

*p*-value from analysis of the variance (ANOVA) and adjusted for energy intake

As shown in Table 2, the highest FQS group consumed significantly more vitamins such as B12, E and C, fruits, nuts and legumes, vegetables, yogurt, and whole grains (*P* < 0.05). Furthermore, the greater FQS group consumed fewer red meat, refined grains, sugar-sweetened beverages, desserts and ice cream, potato and fried food (*P* < 0.05). However, there were no significant differences in vitamins such as B6, and thiamin, potato chips, and coffe consumption between the lowest and highest FQS groups (*P* > 0.05).

### BM anti-oxidant and infant urinary anti-oxidant by tertiles (T) categories of FQS (Table 3)

**Table 3:** BM anti-oxidant and infant urinary anti-oxidant by tertiles (T) categories of FQS

Variables	Tertiles of FQS			*P* value^a^
	**T1** 118(33.71%)	**T2** 122(34.85%)	**T3** 110(31.42%)	
Milk DPPH ( µmol Trolox equivalent /L)	314 ± 102	319 ± 70	325 ± 88	0.05**
Milk FRAP (µmol /L)	526 ± 152	554 ± 146	564 ± 156	0.26
Milk MDA (μmol TBARs/L)	0.15 ± 0.06	0.13 ± 0.09	0.14 ± 0.07	0.34
Milk Thiol (μmol/L)	75.4 ± 18.6	77.7 ± 18.9	81.9 ± 23	0.01*
Milk Calcium ( mg/dL)	8.82 ± 1.05	8.90 ± 1.09	9.13 ± 1.21	0.001** ^**b**^
Milk Protein (g/dL)	1.29 ± 1.03	1.63 ± 1.21	1.71 ± 1.17	0.02**
Milk Triglyceride (mg/dL)	4.52 ± 1.09	4.14 ± 1.09	3.94 ± 1.07	0.17
Infant urinary FRAP (µmol /L)	18.2 ± 16	22.5 ± 14	23.2 ± 17	0.05***
Infant urinary DPPH (mmol eq. Trolox/L)	8.6 ± 7.7	10.7 ± 8.1	10.5 ± 7.3	0.04***
Infant urinary MDA (μmol TBARs/L)	1.26 ± 1.4	1.80 ± 1.8	1.82 ± 1.6	0.15

Diphenylpicrylhydrazyl (DPPH), Ferric reducing ability of plasma (FRAP), Malondialdehyde (MDA)

^a^*p*-value from analysis of the variance (ANOVA)

^b^Significant after even Bonferroni correction

^*^1–2 *P* < 0.05

^**^2–3 *P* < 0.01

^***^1–3 *P* < 0.001

Milk DPPH, thiol, calcium, protein, and infant urine DPPH and FRAP levels were significantly elevated in the highest tertile of the FQS than in the lowest tertile (*P* < 0.05; Table 3).

### Multivariable adjusted β (95% CIs) for content in BM and infant urinary across tertiles of FQS (Table 4)

**Table 4:** Adjusted linear regression analysis (β, 95% confidence intervals) for content in BM and infant urinary across tertiles of FQS

**Tertiles of FQS**	Milk DPPH β(95% CI)	Milk Thiol β(95% CI)	Milk Calcium β(95% CI)	Milk Protein β(95% CI)	Infant urinary FRAP β(95% CI)	Infant urinary DPPH β(95% CI)
T2 vs T1	0.09(0.00 to 0.18)	0.046(-0.057 to 0.143)	0.69(0.65 to 0.74)*	0.08(0.06 to 0.11)*	0.01 (-0.09 to 0.10)	0.020(-0.018 to 0.059)
T3 vs T1	0.26(0.07 to 0.48)*	0.005(0.001 to 0.009)*	0.88(0.83 to 0.89)*	0.09(0.07 to 0.012)*	0.003(0.002 to 0.005)*	0.053(0.015 to 0.093)*

Tertile 1 (lower adherence) was considered as reference group

Breast milk contents were adjusted for maternal age, maternal BMI and energy intake

Infant urine contents were adjusted for infant age

^*^p < 0.05

Linear regression analysis demonstrated that third FQS tertile was associated with higher milk DPPH (β = 0.26; 95% CI: 0.07 to 0.048), thiol (β = 0.005; 95% CI: 0.001 to 0.009), calcium (β = 0.88; 95% CI:0.83 to 0.89), protein (β = 0.09; 95% CI:0.07 to 0.012) and infant urinary FRAP levels (β = 0.003; 95% CI: 0.002 to 0.005) and DPPH levels (β = 0.053; 95% CI:0.015 to 0.093) versus first FQS tertile.

## Discussion

We have investigated the association between maternal diet quality and BM nutrient content as well as TAC of infant urine. A significant positive association was found between maternal FQS and quality indicators of BM including DPPH, thiol, calcium, and protein. A higher FQS was also associated with higher TAC levels in the infant's urine, indicating the important role of maternal nutrition in the proper development of the infant. There is increasing research on the effect of maternal dietary patterns during pregnancy and lactation on BM composition as well as the anthropometric indices of the offspring [37–39]. Hu and colleagues recently concluded that following diet patterns rich in animal and plant foods leads to higher fat and lower protein content in colostrum. Whereas in mature milk, animal and plant foods-rich dietary patterns were associated with increased carbohydrates and decreased fat content, respectively [25]. According to Lisa and colleagues, Indonesian mothers with multiple micronutrient deficiencies had lower content of B vitamins, retinol, sodium, copper, and iron in their BM. In addition, concentrations of vitamin B12, beta-cryptoxanthin, retinol, selenium, and iron were positively correlated with the maternal nutritional condition at 5 months postpartum [40, 41]. Huang et al. have reported that consuming more red meat, grains, and eggs during lactation resulted in higher content of total dry matter, protein, and energy in BM [42].

Calcium concentration is one of the most important indicators of BM quality due to its important role in ossification and cellular signaling [43]. There are obvious differences between countries in calcium concentration of BM, which can be due to variations in dietary guidelines of different geographical areas. However, in most of the studies, no correlation was found between maternal dietary calcium/vitamin D intake and BM calcium content [44, 45]. Nevertheless, based on our findings, regardless of cultural and geographical differences, the quality of maternal nutrition appeared to be positively correlated with BM calcium content.

Another indicator of BM quality is protein content, which is used as sources of nitrogen, amino acids, and antioxidant peptides. BM proteins are also involved in increasing the bioavailability of micronutrients, strengthening the immune system and non-immunological defenses, and forming microbiomes [46]. Studies have shown that the protein content of BM varies greatly between individuals and is influenced by habitual maternal intakes [47, 48]. Debski et al. have reported that the BM’s protein concentration in vegetarian and non-vegetarian mothers was 10.2 g/ml and 9.9 g/100 ml, respectively [49]. In contrast, Huang and colleagues reported a negative correlation between protein concentrations in BM and a greater adherence to a pattern of high intake of vegetables, legumes, and low intake of poultry, red meat, and eggs [50]. Therefore, the increase in BM protein in response to the increase in maternal FQS is in line with previous evidence.

One of the major macronutrients in BM is fat, about 98% of which is in the form of triglycerides [51]. BM fat is not only the main source of energy for newborns but also participates in the synthesis of prostacyclin precursors, thereby improving ventricular function and the architecture of membrane-rich tissues [52]. Several studies have shown that the fat content of BM, especially long-chain polyunsaturated fatty acids (LC-PUFAs), is significantly affected by the dietary pattern of lactating mothers [53, 54]. It has been found that approximately 75% of linoleic acid in BM is obtained directly from the maternal diet [55, 56]. In addition, it has been shown that the concentration of medium-chain fatty acids (MCFAs) in BM is also affected by the fat and carbohydrate intakes from the mother's diet [57]. However, we did not find a significant relationship between maternal diet quality and triglycerid content of BM. The antioxidant level of the BM is another potential indicator of diet quality, that may pave the way for health promotion through diet-based approaches [58].

According to evidence from previous studies, the antioxidant content of transitional and mature milk as well as infant urine may be related to the mother's dietary intake of vitamins A, E, and C, beta-carotene, and vegetables during pregnancy and lactation [59–63]. Significant correlations between maternal nutrition quality and TAC levels of BM and infant urine are evident from our results, too. We used the most well-known analytical methods to study the antioxidant status of biological samples, including FRAP, DPPH, TBARs, and Ellman’s assays, which are simple, inexpensive, and fast. Using FRAP and DPPH methods, our team reported a much higher TAC in colostrum compared to transitional and mature milk, as well as a significant relationship between BM's antioxidant capacity and maternal plasma [64].

In this study, total antioxidant status of BM and infant urine samples was determined using four different assays. Oxidative stress is related to an imbalance between free radical synthesis and antioxidant defenses, and it is often essential to evaluate the counterpart of oxidation, the total antioxidant status. Additional biomarkers have pointed out related antioxidant status/capacity status, rather than OS. As antioxidants can act additively or synergistically, they are absorbed and used in the human body in different ways, and the evaluation of total antioxidant activity yields more reliable data compared to the measurement of one antioxidant individually. These include indices that reflect the total scavenging potency of a plasma, serum, breast milk or urine aliquot, following, for instance, the addition of a radical-forming compound. Currently, the most often used tests for the evaluation of antioxidant stress are the and DPPH assays. The TBARs and Ellman’s assays were used to evaluate the lipid peroxidation and the free thiol groups, respectively.

We have assessed a wide spectrum of potential confounders in our study. Steps of sampling and data analysis were conducted with optimal quality control. However, this study has some limitations; the dietary assessment tool was developed for the general population and might not be completely appropriate for breastfeeding women, therefore our findings should be interpreted with caution. Also, due to the cross-sectional nature of the present research design, no definite causality can be inferred from the results. Therefore, a more accurate understanding of the role of maternal diet quality in BM composition requires more studies. Findings from such research can suggest effective strategies for nutritional interventions in lactating women.

## Conclusion

We found that the quality of the mother's diet (indicated by FQS) had a signficant association with BM composition and the infant's urine. Further studies are necessary to confirm these results using a larger population sample.

## Data Availability

The datasets used and/or analysed during the current study are available from the corresponding author on reasonable request. (The presented results are part of a comprehensive study with a large amount of unpublished data, the full content of which is not possible.)

## Abbreviations

FQS: Food quality score
BM: Breast milk
FRAP: Ferric reducing antioxidant power
DPPH: 2, 2′-Diphenyl-1-picrylhydrazyl
TBARS: Thiobarbituric acid reactive substances
TAC: Total antioxidant capacity
SIDS: Sudden Infant Death Syndrome
CVD: Cardiovascular disease
IgA: Immunoglobulin A
ROS: Reactive oxygen species
FFQ: Food frequency questionnaire
TPTZ: Ferric-tripyridyl-s-triazine
TBA: Thiobarbituric acid
MDA: Malondialdehyde
DTNB: 5,5’-Dithio-bis-(2-nitrobenzoic acid)
SBP: Systolic blood pressure
DBP: Diastolic blood pressure
LC-PUFAs: Long-chain polyunsaturated fatty acids
MCFAs: Medium-chain fatty acids
